# Effects of l-ascorbic acid on growth, non-specific immunity, antioxidant capacity, and intestinal and hepatopancreatic histology of red swamp crayfish, *Procambarus clarkii*

**DOI:** 10.1038/s41598-023-48609-0

**Published:** 2023-12-05

**Authors:** Hala S. Khalil, Hamdy Omar Ahmed, Nehal Elkhouly, Mohammed F. El Basuini, Asmaa M. El-Nokrashy, Amira A. A. Hessein, Asmaa A. Khaled, Amr M. A. Rashad, Mohamed Kord, Diaa Alkenawy, Mohsen Abdel-Tawwab, Hany M. R. Abdel-Latif

**Affiliations:** 1 Aquaculture Department, Faculty of Fish Resources, Suez University, Suez, 43221 Egypt; 2 College of Fisheries and Aquaculture Technology, Arab Academy for Science, Technology, and Maritime Transport, Alexandria, Egypt; 3 National Institute of Oceanography and Fisheries (NIOF), Cairo, Egypt; 4 Animal Production Department, Faculty of Agriculture, Tanta University, Tanta, 31527 Egypt; 5 Faculty of Desert Agriculture, King Salman International University, El Tor, South Sinai 46618 Egypt; 6 Department of Aquaculture, Faculty of Aquatic and Fisheries Science, Kafrelsheikh University, Kafr Elsheikh, Egypt; 7 Department of Fish Nutrition and Feed Technology, Central Laboratory for Aquaculture Research, Agriculture Research Centre (ARC), Abbassa, Abu Hammad, Sharkia Egypt; 8 Animal and Fish Production Department, Faculty of Agriculture (Saba Basha), Alexandria University, Alexandria, 21531 Egypt; 9 Animal and Fish Production Department, Faculty of Agriculture (El-Shatby), Alexandria University, Alexandria, 21545 Egypt; 10 Central Laboratory for Agricultural Climate, Agriculture Research Center (ARC), Giza, Egypt; 11 Limnology Department, Central Laboratory for Aquaculture Research (CLAR), Agriculture Research Center (ARC), Abbassa, Abo-Hammad, Sharqia Egypt; 12 Department of Fish Biology and Ecology, Central Laboratory for Aquaculture Research, Agriculture Research Center (ARC), Abbassa, Abo-Hammad, Sharqia Egypt; 13 Department of Poultry and Fish Diseases, Faculty of Veterinary Medicine, Alexandria University, Alexandria, 22758 Egypt

**Keywords:** Cell biology, Immunology, Physiology, Structural biology, Zoology

## Abstract

This study investigated the dietary effects of coated l-ascorbic acid (LA) on growth, feed utilization, survival, serum biochemical indices, immunity, antioxidant capacity, and intestinal and hepatopancreatic histology of the pre-adult red swamp crayfish. Four isoproteinous and isolipidic diets were formulated to contain several LA levels as 0, 1300, 1600, and 1900 mg/kg and designated as control (LA0), LA13, LA16, and LA19, respectively. However, the analyzed LA concentrations in diets were 0.00, 199.57, 360.45, and 487.50 mg/kg in LA0, LA13, LA16, and LA19, respectively. Triplicate treatments of crayfish (21.60 ± 0.14 g) were fed the test diets and reared in fiberglass tanks with a density of 20 individuals per each for eight weeks. Results revealed that all LA treatments had significantly enhanced growth performance compared to the control. Of interest, the LA16 treatment recorded the highest final tank biomass, biomass gain, total feed intake, condition factor, and muscle yield among the other treatments. The tank feed conversion ratio was significantly decreased in LA treatments compared to the control. Moreover, dietary LA16 and LA19 had significantly higher survival rates (93.3%) compared to (85.0%) in the LA0 group. All dietary doses of LA significantly increased serum parameters (total protein, albumin, globulin, lysozyme activity) and respiratory burst activity compared to the LA0 treatment. Dietary LA16 significantly boosted the hepatopancreatic antioxidant capacity, manifested by decreased malondialdehyde concentrations, increased catalase, superoxide dismutase, and glutathione peroxidase enzyme activities, and reduced glutathione content compared to the LA-free diet. A normal histoarchitecture of the hepatopancreatic tubules was found in all LA treatments except with some minor degenerative changes in the tubular lumen, and hepatopancreatic cells associated with enlarged nuclei were found in the LA19. However, normal intestinal histoarchitecture was found in all treatments with no recorded intestinal lesions. Of interest, the polynomial regression performed on the analyzed LA concentrations suggested that 380 mg/kg would be suitable to provide maximal biomass gain for pre-adult crayfish. In conclusion, results revealed that coated LA could enhance the growth, immunity, and antioxidant capacity of pre-adult red swamp crayfish, suggesting its potential as a functional and necessary micronutrient for crayfish diets.

## Introduction

Aquaculture has been greatly developed in many countries worldwide to provide human beings with nutritious proteinous food from diverse fish and crustacean species^1–3^. Nowadays and in the coming years, with the continuously elevated fish prices that are associated with world inflation, researchers have directed toward finding new and cost-effective fish or crustacean species to be farmed to find new proteinous sources for human beings^4,5^. Red swamp crayfish (*Procambarus clarkii*) is a freshwater crustacean that has a natural distribution in the south-central part of America and northern Mexico, but it has been introduced to several countries, where it may become an invasive species^6^. It was farmed in China and then distributed to other geographic areas. In the 1980s, it was introduced into Egypt as an exotic species, and it spread to the river Nile and its tributaries and lakes^7^. The red swamp crayfish is characterized by its delicious taste, high muscle protein, and other nutritional attributes that give this species a high market value in various countries across the globe^8^.

In aquaculture, the nutritional supplementation of vitamin C, particularly its traditional form, l-ascorbic acid (LA), has gained particular attention in the last decade because of its vital importance for farmed aquatic organisms^9^. Awareness was given to LA because of its well-known functionality and health benefits as a necessary micronutrient for improving the health status and physiological responses of many finfish and crustacean species^10^. Notably, it was reported that aquatic organisms differ in their LA requirements from livestock animals as animals can create LA from glucuronic acid, while finfish and crustaceans have a deficit of the l-gulonolactone oxidase enzyme required for LA biosynthesis^11^. This makes them depend mainly on a constant supply through their formulated diets^12^. On the other hand, LA is a water-soluble vitamin and sensitive to being degraded and losing its functionality (and bioactivity) during the processing and storage of the formulated aquafeed. Moreover, leaching losses may also occur in LA and most water-soluble vitamins^13^. Thus, researchers conducted several studies to find alternative LA forms to keep its activities in aquafeed, such as using coated LA^14,15^ or its derivatives^10,16^.

Generally, LA is necessary for all aquatic animals for growth, reproduction, wound healing, immunity, and disease resistance^9^. It is also required for the biosynthesis of collagen and several hormones and has an essential role in mitigating the toxic impacts of pollutants^17^. For red swamp crayfish, dietary LA is vital for growth, immunity, and reproductive performance^18^. In shrimps, it was found that dietary LA deficiency has led to poor growth and feed conversion, dropped molting frequencies, incomplete molting cycle, exoskeleton lesions, reduced stress tolerance, impaired disease resistance, and elevated mortalities^14^.

The nutritional requirement of LA differs among crustaceans. For example, it was shown that supplemental LA (100 mg/kg) promoted the growth, antioxidant capacity, and digestive enzyme activities of freshwater prawn (*Macrobrachium malcolmsonii*)^19^. However, 130 mg/kg of ascorbic acid 2-monophosphate was optimal for Whiteleg prawn (*Penaeus vannamei*) in the early post-larval stages of life^20^. Hence, its efficacy is also associated with the supplementation dose, forms, and life stages of aquatic animals^9^. Several factors should be considered to optimize the LA requirements for fish and crustaceans, such as differences in requirements among species^21^, size and developmental stage^12,22^, LA forms^23^, among several others. Hence, all these factors should be considered during the supplementation of LA in diets prepared for fish and crustaceans.

A few research studies were conducted to evaluate LA effects on red swamp crayfish. Kong et al.^15^ clarified that coated LA (265.67 mg/kg) was optimal for the maximum growth of *P. clarkii* juveniles (7.0 g body weight). Another study found that dietary l-ascorbyl-2-monophosphate (300.95 mg /kg) was optimal for *P. clarkii* juveniles (0.7 g body weight)^16^. This study aims to determine the impacts of supplemental LA levels on the performances of pre-adult *P. clarkii*. Thus, its effects on growth, survival, muscle composition, hemolymph biochemical indices, non-specific immune responses, oxidative stress biomarkers, and intestinal and hepatopancreatic histology were investigated.

## Material and methods

### Ethical consent

Experiments accomplished in the existing research have been licensed by the Local Experimental Animal Care Committee, Faculty of Agriculture, Alexandria University. Ethical Consent was obtained from the Institutional Animal Care and Use Committee at Alexandria University with Approval Code (AU-08/22/12/28/3/121). All methods in this study were performed in accordance with the relevant guidelines and regulations and ARRIVE guidelines (https://arriveguidelines.org).

### Preparation of the experimental diets

Coated vitamin C (l-Ascorbic Acid; LA), with a purity of ≥ 99.0% crystalline powder, was commercially obtained from Sigma-Aldrich^®^ agent (Egypt). Four isoproteinous and isolipidic diets were formulated, whereas the basal diet was supplemented with different LA levels as 0, 1300, 1600, and 1900 mg/kg and labeled as LA0 (control), LA13, LA16, and LA19, respectively. The raw dietary ingredients were thoroughly crushed into fine particles to prepare the test diets and then strained via a mesh sieve. The chosen LA levels were added to the diets at the expense of microcrystalline cellulose. The LA levels were mixed with the raw diet components. A suitable amount of water and oil was added to each kg feed during the mixing procedures to moisten the diets and get a dough. The generated dough was then passed across a meat mincer to form pelletized diets (2 mm diameter). The formulated test diets were dried in the air, preserved in Ziplock bags, and kept in a refrigerator (− 20 °C) until utilized. The LA concentrations in the formulated LA-supplemented diets were analyzed directly by the high-performance liquid chromatography (HPLC) method, as explained by Shiau and Hsu^24^ and Wang et al.^25^. The analyzed LA concentrations of the test diets were 0.00, 199.57, 360.45, and 487.50 mg/kg in the LA0, LA13, LA16, and LA19 diets, respectively.

### Chemical (nutrient) compositions of the test feeds

Information about the feed formulations, ingredients, and chemical compositions of the test feeds is given in Table 1. The analysis was conducted in line with the Association of Analytical Chemists (AOAC) procedures^26^. Crude protein (CP; %) was examined using Kjeldahl’s method for nitrogen determination. Crude fiber (CF; %) was evaluated according to the Weende method, which is based on the digestion of non-cellulosic compounds by sulfuric acid and potassium hydroxide. Ash (%) levels were assessed after the sample burning at 550 °C in a muffle furnace for one day. Moisture (%) content was assessed after the sample drying to a constant weight at 105 °C. Crude lipid (CL; %) content was evaluated by ether extraction using a Soxtec apparatus System.

**Table 1:** Ingredients and proximate chemical composition (%) of the experimental diets supplied with various l-ascorbic acid (LA) levels.

Feed ingredients	Diets with different LA levels			
	LA0 (Control)	LA13	LA16	LA19
Fish meal (FM)⁕	10.0	10.0	10.0	10.0
Soybean meal (SBM)^#^	35.0	35.0	35.0	35.0
Corn gluten meal (60% CP)	4.8	4.8	4.8	4.8
Rice bran (14% CP)	11.50	11.5	11.5	11.5
Yellow corn meal	14.5	14.5	14.5	14.5
Wheat bran	9.0	9.0	9.0	9.0
Wheat flour	12.0	12.0	12.0	12.0
Sunflower oil	0.70	0.70	0.70	0.70
Microcrystalline cellulose	1.70	1.57	1.54	1.51
l-ascorbic acid^a^	0.0	0.13	0.16	0.19
Vitamin and mineral premix ^b^	0.30	0.30	0.30	0.30
Dicalcium phosphate^b^	0.50	0.50	0.50	0.50
Total	100	100	100	100
Proximate chemical analysis (% on DM basis)				
Dry matter (DM)	89.64	89.65	89.65	89.65
Crude protein (CP)	30.00	30.01	30.02	30.02
Crude lipids (CL)	5.8	5.8	5.8	5.8
Ash	5.91	5.92	5.92	5.92
Crude fiber (CF)	5.31	5.31	5.31	5.31
Nitrogen free extract (NFE)^c^	52.98	52.95	52.95	52.94
Analyzed LA (mg/kg)	0.00	199.57	360.45	487.50

*Danish FM (72% CP), obtained from TripleNine Fish Protein, DK-6700 (Denmark).

^#^Egyptian soybean flour (44.8% CP), obtained from Cargill Trading Egypt Co. (Egypt).

^a^l-ascorbic acid (LA), with a purity of ≥ 99.0%, crystalline powder, purchased from Sigma-Aldrich® agent (Egypt).

^b^Purchased from AGRI-VET for Manufacturing Vitamins and Feed additives (10th of Ramadan City A2, Egypt). Vitamin and Mineral composition (per kg premix mixture): Vitamin premix including 700 mg (vitamin B1); 3500 mg (vitamin B2); 1000 mg (vitamin B6); 7 mg (vitamin B12); 8,000,000 IU (vitamin A); 2,000,000 IU (vitamin D3); 7000 mg (vitamin E); 1500 mg (vitamin K3); 50 mg (biotin); 700 mg (folic acid); 20,000 mg (nicotinic acid), and 7000 mg (pantothenic acid). Mineral premix including 40 g (Zinc); 20 g (Iron); 2.7 g (Copper); 0.34 g (Iodine); 53 g (Manganese); 70 mg (Selenium); 70 mg (Cobalt) and Calcium carbonate as carrier up to 1 kg.

^c^NFE = 100 − (CP + CL + CF + Ash).

### Experimental animals: Collection, acclimation, and rearing conditions

Pre-adult red swamp crayfish were collected from fertilized ponds at WorldFish Center, Abbassa, Egypt. Animals were transferred into the rearing tanks and then left for acclimatization to the new environments. Crayfish were fed a pelleted basal diet (LA0; control diet without supplements) during a 2-week adaptation period. Following the acclimatization, 240 crayfish (ca 21 g in weight and 6.5 cm initial length) were bulk weighed in groups of 20 and transferred to fiberglass tanks (120 cm × 80 cm; water depth 30 cm) to create triplicates for each feed treatment (LA0, LA13, LA16, and LA19). Crayfish were assembled, bulk-weighed, and allotted into an indoor system in the Wet Laboratory belonging to the WorldFish Center, Abbassa, Egypt. Each tank was supported with 4 PLA water pipes to act as a shelter for raising crayfish and to prevent cannibalism and predation. Animals were deprived of feeding on the basal diet for one day (24 h) before starting to feed on the test diets. During the trial, crayfish were hand-fed on the corresponding formerly prepared test diets to apparent satiety three times daily (08.00 a.m., 12.00 a.m., and 3.00 p.m.) for eight weeks (56 days). The quantity of feed ingested per tank was recorded daily. Moreover, the leftover meal was garnered by siphoning 2 h post-feeding and then oven-dried at 60 °C to accurately evaluate the tank feed intake. During the feeding trial, the water in each tank was kept at a depth of 30 cm with constant aeration to preserve the water quality adequate for rearing crayfish. Crayfish in all rearing tanks were examined daily throughout the feeding period, and dead animals were counted, recorded, and then discarded using complete hygienic disposal methods. Siphoning was conducted every two days, and water renewal was performed by exchanging 1/3 of the water volume and replacing it with new, well-aerated, de-chlorinated tap water. The siphoning was performed to remove feces and newly molted shells before offering a new feed. The water quality indices were monitored weekly, and their average values were sustained at 26.50 ± 0.5 °C for temperature, 6.5 ± 0.03 for pH, 7.5 ± 1.0 mg/L for dissolved oxygen, and 0.067 ± 0.033 mg/L for ammonia-N. The lighting regime was an 8 h light to 16 h dark cycle.

### Sampling procedures

Animals in all treatments were starved for 24 h after the feeding trial ended. Before the sampling procedures, crayfish were subjected to hypothermic anesthesia via compression in an ice tank for 15 min^27^.

#### Hemolymph sampling

The hemolymph samples were obtained directly after the piercing of the heart of the crayfish. Three hemolymph samples (n = 3) were taken from each treatment. In this step, an equal volume of hemolymph from three crayfish per tank was pooled, homogenously mixed, and considered one sample. Two sets of hemolymph samples were collected using a 1-mL sterile syringe. The first set was collected and pooled from 3 crayfish per tank and was drawn into a sterile Eppendorf tube containing the same volume of the cooled anticoagulant. This sample was used to evaluate the respiratory burst activity (RBA) of the hemocytes. The second set was collected and pooled from another 3 crayfish per tank and was drawn without anticoagulant into a sterile Eppendorf tube and used for serum separation. To collect the serum, hemolymph samples were left on ice for 6 h in a standing position to separate the supernatant. The supernatant samples were then separated after centrifugation at 3000×*g* for 10 min at 4 °C. These samples were collected, pooled, refrigerated, and then stored at − 20 °C until being used to evaluate serum biochemical assays.

#### Tissue sampling

Following the hemolymph sampling procedure, the bloodless crayfish from each tank were placed on ice and then aseptically dissected to get different tissues (hepatopancreas, tail muscle, and intestines). Samples obtained from the tail muscle (n = 3) were weighed to evaluate the muscle yield (%). Further, the tail muscle samples were cryopreserved and held at − 20 °C to assess the proximate chemical composition analysis. The hepatopancreas samples (n = 3) were held at − 20 °C to be frozen before being used to prepare homogenate samples. The homogenate samples were prepared from 100 mg of the thawed hepatopancreas sample, homogenized well in sterile, cold physiological buffer saline (PBS) (at a volume ratio of 1:9) by a tissue homogenizer, and then centrifuged (5000×*g* for 10 min at 4 °C). After centrifugation, sediment was discarded, and the supernatant was collected and used to evaluate the oxidative stress biomarkers. Other hepatopancreas (n = 3) and intestinal samples (n = 3) were washed in sterile PBS and then rapidly fixed in a neutral and freshly prepared 10% formalin solution for histological analysis.

### Evaluation of growth, feed utilization, morphology indices, and survival %

After the end of the feeding duration (56 days), crayfish were deprived of feeding on LA-supplemented diets for 24 h as a period of starvation. This step is essential to remove any ingested food from the gastrointestinal tract (GIT) of crayfish, according to Davis and Robinson^28^. Crayfish from each tank were collected into plastic buckets, counted (to know their final number), and then bulk weighed. The total final biomass, biomass gain, and total feed intake have been estimated. The tank feed conversion ratio was also evaluated as the total tank feed intake split by the tank biomass gain.

To calculate the morphology indices such as muscle yield (%) and condition factor (CF, g/cm^3^), three crayfish were taken from those present in each tank. Then, individual final body length (cm), individual final weight (g), and individual final muscle weight (g) were evaluated. Muscle yield and CF were assessed as follows:

$$ {\text{Muscle yield }}\left( \% \right)\, = \,{1}00\, \times \left[ {{\text{Individual final muscle weight }}\left( {\text{g}} \right)/{\text{Individual final weight }}\left( {\text{g}} \right)} \right]\,. $$

$$ {\text{CF }}\left( {{\text{g}}/{\text{cm}}^{{3}} } \right)\, = \,{\text{Final body weight }}\left( {\text{g}} \right)/{\text{Final body length }}\left( {{\text{cm}}} \right)^{{3}} . $$

To calculate the crayfish survival rate (SR; %), crayfish per tank were totaled and counted. Then, SR (%) was evaluated according to the following equation:

$$ {\text{SR }}\left( \% \right)\, = \,{1}00\, \times \,[{\text{Final number of crayfish per tank/Initial number of crayfish per tank}}]. $$

### Proximate chemical composition of crayfish muscles

The proximate chemical composition of the abdominal muscles of the crayfish from different treatments was conducted in line with the AOAC procedures^26^, whereas CP (%), ash (%), moisture (%), and CL (%) were evaluated as previously described in "Chemical (nutrient) compositions of the test feeds" section.

### Serum biochemical indices

Serum biochemical indices such as alanine aminotransferase (ALT; U/L), aspartate aminotransferase (AST; U/L), total protein (TP; g/dL), albumin (ALB; g/dL), and globulin (GLO; g/dL) were assayed using commercially purchased diagnostic kits (Spinreact, S.A., Gerona, Spain). Serum ALT and AST enzyme activities were measured in line with the method illustrated by Reitman and Frankel^29^. Hemolymph TP concentrations were assessed per the methodology explained by Bradford^30^, with bovine serum ALB as the standard. Hemolymph ALB was estimated in accordance with the method depicted by Doumas et al.^31^. Hemolymph GLO levels were calculated by subtracting ALB from TP content.

### Non-specific immune assays

Serum lysozyme (LZM) activities were assayed by the turbidimetric method, as illustrated by Ellis^32^. Serum LYZ activity was assayed using *Micrococcus lysodeikticus* (Sigma-Aldrich, USA) suspension. One LYZ unit was outlined as the volume of the serum sample causing a reduction in the absorbance of 0.001 per min at 530 nm. The hemocyte respiratory burst activity (RBA) in the hemolymph samples was evaluated by using the nitro blue tetrazolium (NBT) test as a method used to determine superoxide anion production. RBA was then quantified via a spectrophotometer at OD = 630 nm as per the protocol illustrated by Song and Hsieh^33^.

### Oxidative stress biomarkers

The supernatant resultant from centrifugation of the hepatopancreatic homogenate was used for evaluation of the enzymatic antioxidant mechanisms by measuring the superoxide dismutase (SOD), catalase (CAT), and glutathione peroxidase (GPx) enzyme activities. Moreover, the reduced glutathione (GSH) and malondialdehyde (MDA) concentrations were also assayed. The diagnostic kits used for the evaluation of CAT and SOD enzyme activities were commercially purchased from MyBioSource Inc. (San Diego, California, USA) as per the directions provided by the producer and following the methods labeled by Aebi^34^ and McCord and Fridovich^35^. The diagnostic kits used for evaluating GPx activity and MDA and reduced GSH concentrations were commercially purchased from Biodiagnostic Co. (Giza, Egypt) as per the directions provided by the manufacturer. Malondialdehyde (MDA) concentrations were evaluated (as lipid peroxidation end product) in the hepatopancreatic tissues at an OD of 532 nm by thiobarbituric acid substances (TBARS) method as per the assay previously explained by Ohkawa et al.^36^ and Ke et al.^37^. All parameters were measured in triplicates. The enzyme assays were defined as U/ mg protein. Reduced GSH content was described as µmol/mg protein. MDA concentrations were expressed as nmol/mg protein.

### Histological procedures

The intestines and hepatopancreatic tissues of crayfish samples were immersed in 10% buffered formalin solution for 1–2 days, dehydrated in a series of graded ethyl alcohol (75%, 85%, 90%, 95%, 100%), cleared in xylene, and then embedded in paraffin to make paraffin blocks^38^. Ten cross-sections (5 μm thickness) were taken from each sample by using a rotary microtome and then stained with H&E. The histological assessment was performed on several photomicrographs captured of the histological sections using a digital camera (Leica EC3, Germany) joined with a light microscope (Leica DM500). Ten microscopic fields are randomly selected for each sample for evaluation. The shape and size of the hepatopancreatic epithelial cells, tubular lumen, and nuclei were examined. The shape and arrangement of the intestinal epithelial cells, intestinal lumen, and intestinal layers (epithelium, lamina propria, submucosa, and muscularis layer) were examined. The abnormal histological structure and lesions were judged by a professional histopathologist for qualitative evaluation of the results. Abnormal hepatopancreatic structure (epithelial vacuolation, degenerated cells, degenerated tubules, and enlarged nucleus) and intestinal inflammatory signs were recorded.

### Statistical analyses

A one-way ANOVA was used to determine the dietary effects of supplemental LA levels on the measured parameters of crayfish. Data were expressed as means ± SE. Differences between means were examined at a 5% probability level using Duncan’s test as a post hoc test. Moreover, P < 0.05 was considered a statistically significant difference. Linear and quadratic regression analyses were conducted on the crayfish's growth, morphology indices, survival, and muscle chemical composition to assess the relationship between dietary LA levels and the measured parameters. Best fitted Cubic regression using a polynomial model was performed against the analyzed LA levels to determine the optimum level for maximum biomass gain, as clarified by Zeitoun et al.^39^.

## Results

### Effects of LA on growth, feed utilization, and morphology indices

**Figure 1:**
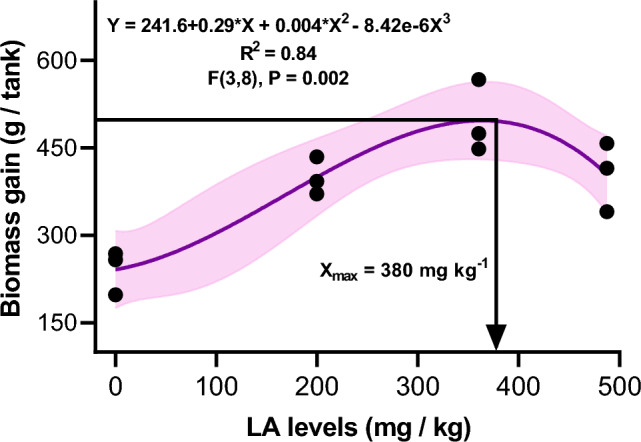
The relationship biomass gain (g/tank) of red swamp crayfish and the analyzed l-ascorbic acid (LA) levels in diets, as described by polynomial regression. Data were expressed as means ± S.E. (n = 3 replicates).

The growth, feed utilization, and morphology indices of crayfish-fed diets supplemented with various LA levels for eight weeks are presented in Table 2. The final tank biomass, biomass gain, and total feed intake were linearly and quadratically increased (P < 0.05) in LA treatments compared to those fed the LA-free diet. Moreover, variations within different treatments showed that their highest levels were noticed in the LA16. The tank FCR significantly decreased in all LA treatments compared to the controls, and LA treatments showed non-significant differences. The morphology indices (CF and muscle yield) and SR (%) were linearly and quadratically increased in LA treatments compared to LA0. Of interest, the muscle yield and CF peaked in the LA16 compared to the other treatments. Furthermore, the highest SR (%) was found in the LA16 and LA19 treatments, with non-significant differences. The polynomial regression analysis of biomass gain and the analyzed LA concentrations suggests that dietary LA of 380 mg/kg would be optimum for maximal biomass gain of pre-adult crayfish (Fig. 1).

**Table 2:** Growth performance, feed utilization, and morphology indices of red swamp crayfish fed diets supplemented with various levels of l-ascorbic acid for 56 days.

Parameters	Experimental groups				PSE^a^	P value	
	LA0	LA13	LA16	LA19		Linear	Quadratic
Initial biomass (g/tank)	433.3	432.0	434.6	434.7	2.84	0.881	0.960
Final biomass (g/tank)	674.9 c	831.6 b	931.5 a	839.2 b	29.90	0.001	0.003
Biomass gain (g/tank)	241.6 c	399.6 b	496.8 a	404.5 b	30.25	0.002	0.004
Total feed intake (g feed/tank)	533.8 c	789.8 b	972.2 a	799.0 b	50.14	0.002	0.003
Tank feed conversion ratio (FCR)	2.21 a	1.98 b	1.96 b	1.98 b	0.041	0.008	0.021
Condition factor (CF; g/cm^3^)	2.24 c	2.81 b	4.04 a	3.12 ab	0.260	0.041	0.058
Muscle yield (%)	9.8 b	11.4 ab	14.1 a	11.1 ab	0.724	0.022	0.056
Survival rate (SR; %)	85.0 c	91.7 b	93.3 a	93.3 a	1.35	0.004	0.020

The values of Means that not sharing a common letter in the same row are significantly statistically different at P < 0.05.

^a^Pooled standard error of the means.

### Effects of LA on proximate composition of crayfish muscles

The effects of dietary LA on the chemical analysis of the crayfish muscles are presented in Table 3. The CP content in the crayfish muscles was linearly and quadratically increased and was significantly elevated in all LA treatments compared with those fed on the LA-free diet (Table 3); however, the CP content in all LA treatments showed non-significant differences between their values (P > 0.05). On the other hand, moisture (%) and CL (%) were decreased linearly and quadratically alongside the increasing dietary LA supplementation. Of interest, the CL (%) was not significantly affected among LA13, LA16, and LA19 treatments (P > 0.05). However, ash (%) was linearly and quadratically elevated in LA16 and LA19 treatments compared to other treatments.

**Table 3:** Proximate chemical composition (% on fresh weight basis) of the muscles of red swamp crayfish fed diets supplemented with various levels of l-ascorbic acid for 56 days.

Parameters	Dietary l-ascorbic acid levels (% feed)				PSE^a^	P value	
	LA0	LA13	LA16	LA19		Linear	Quadratic
Moisture (%)	73.2 a	72.4 ab	71.7 b	71.4 b	0.286	0.019	0.072
Crude protein (CP; %)	13.9 b	15.7 a	15.9 a	15.9 a	0.290	< 0.001	0.002
Crude lipids (CL; %)	7.5 a	6.1 b	6.2 b	6.10 b	0.187	< 0.001	< 0.001
Ash (%)	1.2 b	1.2 b	1.4 a	1.50 a	0.038	0.005	0.001

The values of Means that not sharing a common letter in the same row are significantly statistically different at P < 0.05.

^a^Pooled standard error of the means.

### Effects of LA on serum biochemical indices

**Figure 2:**
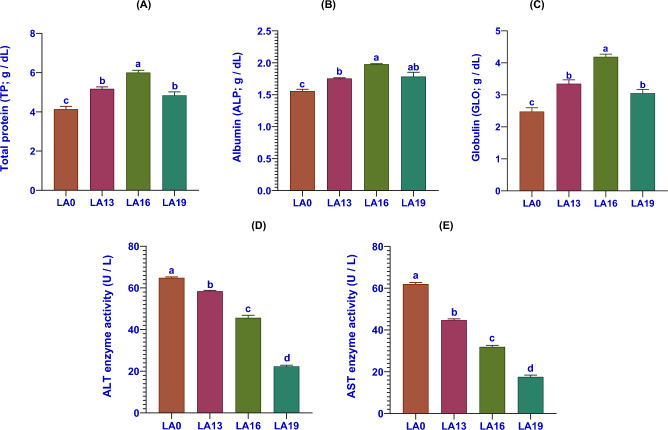
The serum biochemical indices include total protein (g/dL; **A**), albumin (g/dL; **B**), globulin (g/dL; **C**), ALT (U/L; **D**), and AST (U/L; **E**) in red swamp crayfish fed diets supplemented with various levels of l-ascorbic acid (LA) for 56 days (n = 3). Data were expressed as means ± SE. Bars labeled with different letters are statistically significantly different at P < 0.05.

Serum biochemical indices of crayfish fed on LA-supplied diets for 56 days are elucidated in Fig. 2. Compared with the LA0, the TP (Fig. 2A), ALB (Fig. 2B), and GLO (Fig. 2C) contents were significantly increased in all LA treatments. Moreover, their uppermost levels were found in the LA16 compared with the other treatments. Conversely, ALT (Fig. 2D) and AST (Fig. 2E) enzyme activities were significantly decreased in all LA treatments with increasing dietary LA levels in a dose-dependent trend compared to those fed on an LA-free diet.

### Effects of LA on non-specific immunity

**Figure 3:**
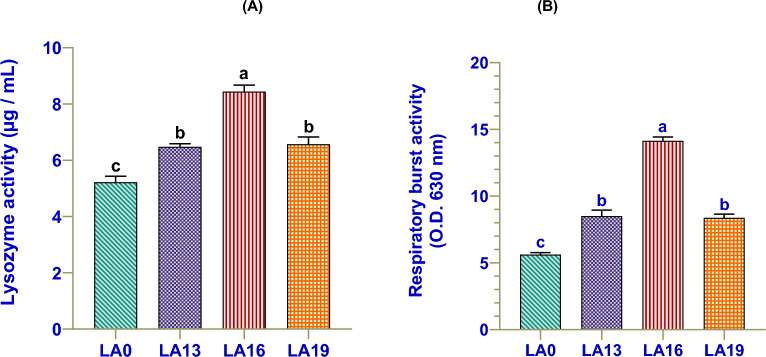
The immune indices include serum lysozyme activity (**A**) and respiratory burst activity (RBA; **B**) of red swamp crayfish fed diets supplemented with various levels of l-ascorbic acid (LA) for 56 days (n = 3). Data were expressed as means ± SE. Bars labeled with different letters are statistically significantly different at P < 0.05.

Dietary LA significantly increased serum LYZ (Fig. 3A) and RBA activities in the hemolymph (Fig. 3B) of crayfish compared with those reared in LA0, and their highest levels were found in LA16. However, their levels showed non-significant differences in the LA13 and LA19 treatments.

### Effects of LA on hepatopancreatic oxidative stress biomarkers

**Figure 4:**
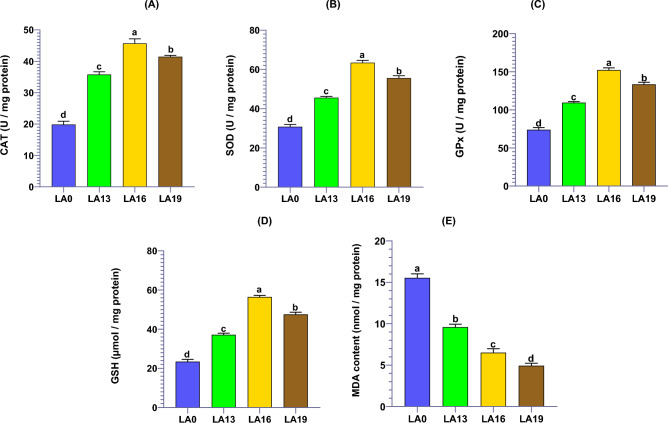
The oxidative stress biomarkers, including CAT (**A**), SOD (**B**), GPx (**C**), reduced GSH content (**D**), and MDA concentration (**E**) in the hepatopancreatic tissues of red swamp crayfish fed diets supplemented with various levels of l-ascorbic acid (LA) for 56 days (n = 3). Data were expressed as means ± SE. Bars labeled with different letters are statistically significantly different at P < 0.05.

The impacts of LA supplementation on the hepatopancreatic MDA concentrations and antioxidant capacity of crayfish are presented in Fig. 4. The enzymatic antioxidant mechanisms, including CAT (Fig. 4A), SOD (Fig. 4B), and GPx (Fig. 4C) enzyme activities as well as the reduced GSH contents (Fig. 4D) of crayfish fed on LA-supplemented diets were significantly increased in response to the dietary LA levels. Furthermore, the highest CAT, SOD, GPx, and GSH levels were found in the LA16. In contrast, their lowest levels were found in LA0 (Fig. 4). The hepatopancreatic MDA concentrations were decreased significantly with regard to dietary LA supplementation levels. Their highest values were found in crayfish fed the LA-free diet (Fig. 4E).

### Effects of dietary LA on hepatopancreatic histology

**Figure 5:**
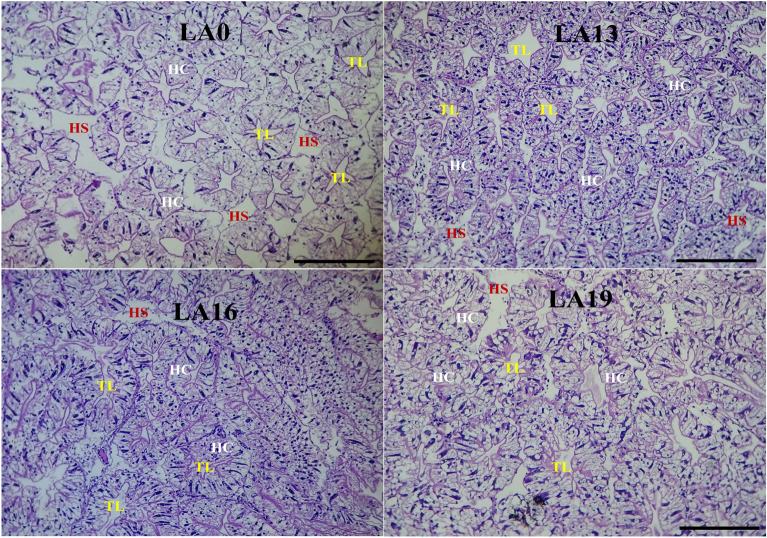
Representative photomicrographs of the hepatopancreatic tubules of red swamp crayfish fed diets supplemented with various levels of l-ascorbic acid (LA) for 56 days. Tissue sections were stained with H & E stain (X 10), scale bar = 20 µm. Hepatic cells (HC), Hemal space (HS), and Tubule lumen (TL).

The impacts of supplemental LA on the hepatopancreatic histology of crayfish in comparison to those reared in control are shown in Fig. 5. The hepatopancreas exhibited well-organized glandular structures and tubular lumens in the LA0, LA13, and LA16 treatments with tightly arranged and intact hepatopancreatic tubules. Moreover, the hepatopancreatic cells (HC) were easily recognized and uniform in shape and size. However, the hepatopancreatic structures in LA19 were characterized by prominent epithelial vacuolation, mildly degenerated hepatopancreatic cells, degenerated hepatopancreatic tubules, lumen dilatation, and an enlarged hepatopancreatic nucleus.

### Effects of LA on intestinal histology

**Figure 6:**
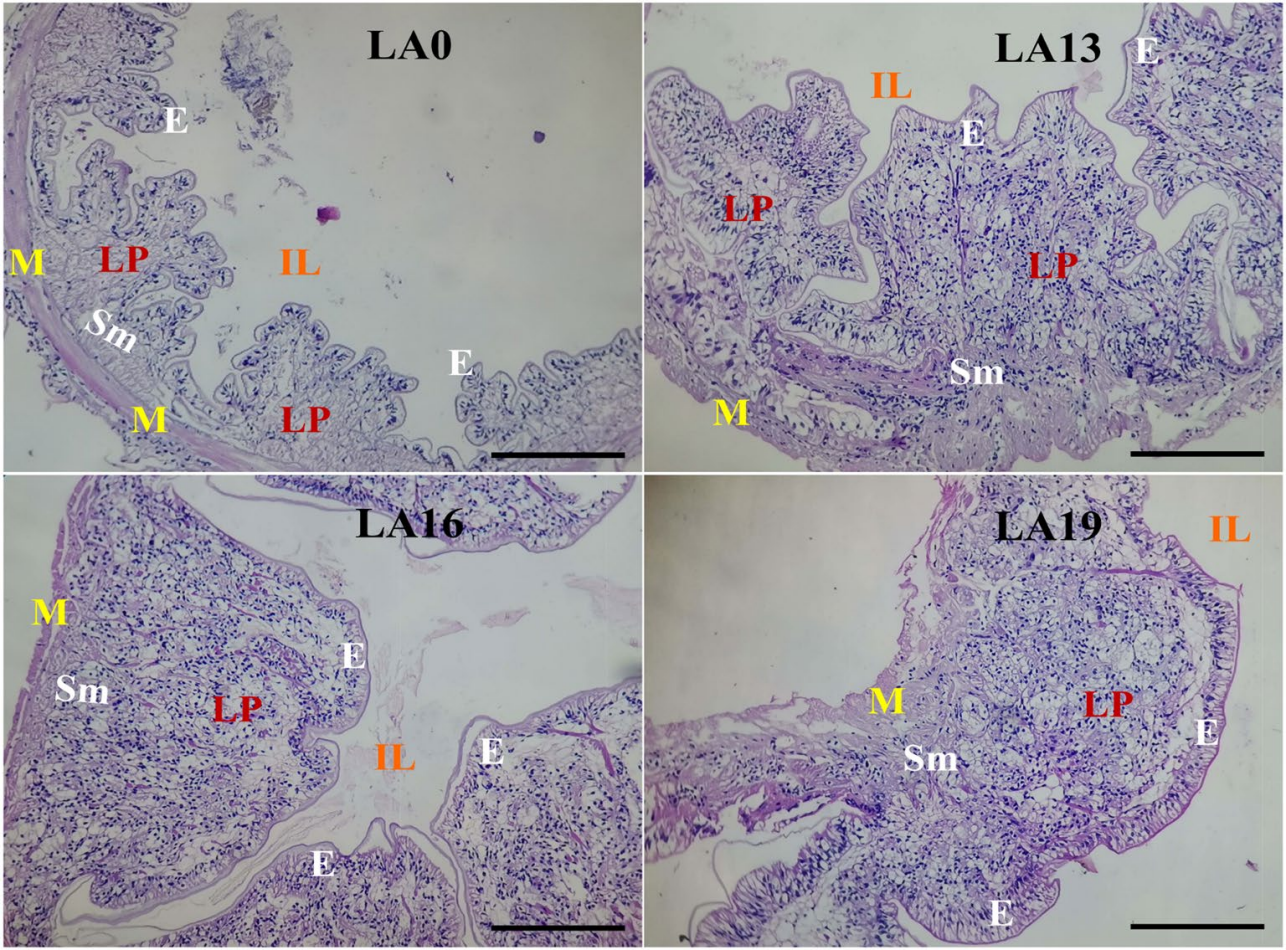
Representative photomicrographs of the intestines of red swamp crayfish fed diets supplemented with various levels of l-ascorbic acid (LA) for 56 days. Tissue sections were stained with H & E stain and observed under light microscopy with (X 10), scale bar = 20 µm. IL: Intestinal lumen; E: Epithelium; LP: Lamina propria; Sm: Submucosa layer; M: muscularis layer.

The intestines of crayfish fed on diets supplied with LA levels in comparison to those reared in the LA0 are shown in Fig. 6. The intestines of crayfish in the LA0, LA13, LA16, and LA19 treatments displayed typical, orderly-organized, and tightly arranged structures of the intestinal lumen, epithelium, lamina propria, submucosa, and muscularis layer with no inflammatory signs recorded.

## Discussion

The beneficial effects of vitamin C (l-ascorbic acid; LA) as a dietary supplement have been proven in aquaculture, with promising results on the health of fish and crustaceans^9^. Herein, LA effects have been investigated on growth, muscle composition, morphology indices, immunity, antioxidant capacity, and intestinal and hepatopancreatic histology of pre-adult *P. clarkii*. The positive effects of LA on growth have been described in various crustacean species, such as the signal crayfish (*Pacifastacus leniusculus*) juveniles^23^, Gazami crab (*Portunus trituberculatus*) juveniles^40^, whiteleg shrimp (*Litopenaeus vannamei*)^41^, giant river prawn (*Macrobrachium rosenbergii*) juveniles^42,43^, *M. malcolmsonii*^19^, and also in *P. clarkii* juveniles^15^. We found linear and quadratic increases in the growth parameters, muscle yield, CF, and SR in all LA-supplemented treatments compared to the controls, and their highest levels were found in the LA16. Vitamin C, a vital micronutrient, is required for enhancing the growth of crustaceans^12^, and its deficiency has led to poor feed conversion, reduced growth, inadequate molting, and high mortality rates^14,24^. The improvement of crayfish survival in the present study could be associated with the positive roles of LA in improving immunity, stress tolerance, and disease resistance^9^. In the present study, the tank FCR decreased significantly in all LA treatments compared to the control. These results were in concordance with those obtained by Kong et al.^15^, who found that dietary LA significantly decreased the FCR alongside increased LA supplementation in red swamp crayfish diets. The optimum dietary LA level in the present study was 380 mg/kg for maximum biomass gain of pre-adult crayfish, as described by polynomial regression. Celada et al.^23^ declared that a 200 mg/kg diet of l-ascorbyl-2-monophosphate-Na is recommended for *P. leniusculus* juveniles. Kong et al.^15^ reported that a 265.67 mg/kg diet is an optimal LA supplementation dose for *P. clarkii* juveniles. Kong et al.^16^ further observed that a 300.95 mg/kg diet is an optimal supplementation dose of l-ascorbyl-2-monophosphate for red swamp crayfish.

Herein, dietary LA significantly increased the CP % in the crayfish muscles in all LA-supplemented treatments compared with those fed on the LA-free diet. The enhancement of CP% in the crayfish muscles may be associated with the dietary roles of LA in the improvement of protein synthesis and amino acid storage, as previously described in the muscles of freshwater prawns^19^. Reports showed that LA could also increase the CP % in the body of *P. clarkii* juveniles^15,16^. However, our results showed that LA-supplementation decreased CL (%) in the crayfish muscles compared to controls. These results differ from those described in *P. trituberculatus* juveniles^40^, *M. malcolmsonii*^19^, and *P. clarkii* juveniles^15^, as these studies reported that dietary LA increased the lipid % in the body of these crustacean species. Indeed, the differences may be linked to several factors, such as LA factors (doses, sources, forms), crayfish factors (crayfish species, initial weights, sizes), experimental conditions, duration of the feeding trial, or others.

Hematology and hemolymph biochemical indices represent crucial bio-indicators for evaluating the health and nutritional state of invertebrates^44^. Total protein (TP) is an important parameter for determining the functional status of crayfish^45^. Moreover, it was previously reported that higher TP, GLO, and ALB contents indicate a vital immuno-competent capacity^27,46^. Results revealed that the TP, ALB, and GLO content significantly elevated with increasing dietary LA levels compared to the controls, indicating that the LA could boost the body immunity of the treated crayfish. ALT and AST are transaminases that contribute to protein metabolism and are sensitive markers for examining hepatopancreatic functions^27,47^. In aquatic organisms, it was found that increased AST and ALT enzyme activities denote dysfunction, necrosis, malfunction, tissue degeneration, and change of protein metabolism in the hepatopancreas^48,49^. Serum ALT and AST activity levels had an inverse relationship with dietary LA, suggesting that high LA in crayfish diets may induce hepatopancreatic injury.

Lysozyme (LZM) is a critical enzyme that exerts antibacterial activities due to its lytic effects on bacterial cell walls^32,50^. It also has a crucial immune function in red swamp crayfish^51^. Respiratory burst activity (RBA) is an essential process that helps hemocytes to get rid of the foreign bacteria from the crayfish hemolymph by phagocytosis by generating reactive oxygen species^33^. Hence, RBA can be regarded as an essential bioindicator to reflect the innate immunity of crustaceans. Herein, it was observed that dietary LA boosted LYZ activity and RBA compared with those reared in the LA0. These results, thereby, reflect the roles of LA in enhancing the immune defense of crustaceans, as previously reported in the giant tiger prawn (*Penaeus monodon*)^52^ and red swamp crayfish^15^. In finfish species, reports also showed that dietary LA could increase LYZ and RBA activity^53,54^.

In aquatic organisms, SOD, CAT, GPx, and reduced GSH are effective antioxidant enzymatic defense mechanisms against free radicals, and they can protect organisms against oxidative stress damage^55,56^. MDA is regarded as the end-product of lipid peroxidation^57^ and an essential marker for assessing oxidative damage^58^. In the present study, CAT, SOD, GPx, and reduced GSH content in the hepatopancreatic tissues of crayfish fed on LA-supplied diets were increased in response to the dietary LA supplemental levels. On the other hand, hepatopancreatic MDA concentrations were decreased with regard to dietary LA supplementation levels. These results suggest the capability of dietary LA to boost the antioxidant capacity of the treated crayfish, reflecting the potent antioxidant properties of dietary LA^59^. Hence, LA can be considered an effective antioxidant and potent free radical scavenger. Previous research has proved the roles of dietary LA in improving the antioxidant capacity of many crustacean species, such as *L. vannamei*^60^, *M. malcolmsonii*^19^, and red swamp crayfish^15^. Similar findings were also reported in several finfish species^61,62^.

Histological analysis is a practical tool for evaluating the nutritional status and health conditions of finfish and crustaceans^63,64^. The present study showed that hepatopancreatic tissues exhibited well-organized glandular structures and tubular lumens in the LA0, LA13, and LA16 treatments with tightly arranged and intact hepatopancreatic tubules. In addition, the hepatopancreatic cells were easily recognized and reasonably uniform in shape and size. This means that dietary LA up to 1600 mg/kg level could be safe and optimum for the typical structures of the hepatopancreatic tissues. On the other hand, LA 1900 mg/kg resulted in prominent epithelial vacuolation, mildly degenerated hepatopancreatic cells, degenerated hepatopancreatic tubules, lumen dilatation, and enlarged hepatopancreatic nucleus. This result was also confirmed by the ALT and AST enzyme activity levels in the present study and their relationship with dietary LA levels. However, the intestines of crayfish in all treatments displayed typical, orderly-organized, and tightly arranged structures of the intestinal lumen, epithelium, lamina propria, submucosa, and muscularis layer, with no inflammatory signs recorded. This indicates that dietary LA did not negatively affect the histology of the crayfish intestines. To the best of our knowledge, no previous results were published on the dietary effects of LA on the histology of red swamp crayfish. Nonetheless, it was reported that dietary supplementation with a 100 mg/kg diet of magnesium l-ascorbyl-2-phosphate improved the histological structure of the hepatopancreas of *P. monodon* juveniles^65^. In finfish, Ibrahim et al.^62^ found that dietary LA improved Nile tilapia's hepatopancreatic histoarchitecture and intestinal histomorphology. Moreover, Yusuf et al.^66^ declared that dietary LA maintained the integrity of the liver morphology of largemouth bass. The positive effects obtained in the present study may be linked with the antioxidant effects of LA, which help to protect the tissues of the treated crayfish from the adverse effects of free radicals.

## Conclusions and future perspectives

The present study's findings revealed that supplemental LA significantly improved growth, feed utilization, survival, enhanced immunity, and hepatopancreatic antioxidant capacity and maintained the normal guts of pre-adult red swamp crayfish. Besides, the treatment fed a diet with 1600 mg/kg LA induced the best non-specific immunity and antioxidant capacity. The polynomial regression analysis revealed that 380 mg/kg of the analyzed LA was optimal for the maximum biomass gain of pre-adult red swamp crayfish. To put it briefly, LA could be considered a crucial micronutrient for enhancing the aquafeed formulated for the crayfish industry. In a theoretical laboratory-based experiment, the aforementioned findings present reference data for improving the ration formulation and health status of crayfish culture. However, the impacts of dietary LA on disease resistance and intestinal microbiome at molecular and nutrigenomic levels warrant further investigations and research studies.

## Data Availability

The datasets used and/or analyzed during the current study are available from the corresponding author on reasonable request.
